# Ghrelin's Effects on Proinflammatory Cytokine Mediated Apoptosis and Their Impact on *β*-Cell Functionality

**DOI:** 10.1155/2015/235727

**Published:** 2015-07-16

**Authors:** Antonia Diaz-Ganete, Gloria Baena-Nieto, Isabel M. Lomas-Romero, Jose Francisco Lopez-Acosta, Irene Cozar-Castellano, Francisco Medina, Carmen Segundo, Alfonso M. Lechuga-Sancho

**Affiliations:** ^1^Research Unit, Puerta del Mar University Hospital, 11009 Cadiz, Spain; ^2^Department of Endocrinology and Nutrition, Jerez de la Frontera General Hospital, 11407 Jerez de la Frontera, Spain; ^3^Andalusian Cellular Reprogramming Laboratory, 41092 Sevilla, Spain; ^4^Genetics and Molecular Biology Research Institute, University of Valladolid-CSIC, 47003 Valladolid, Spain; ^5^Salus Infirmorum Faculty of Nursing, Cadiz University, 11001 Cadiz, Spain; ^6^Department of Maternal and Pediatric Medicine and Radiology, Pediatrics Unit, Puerta del Mar University Hospital, 11009 Cadiz, Spain

## Abstract

Ghrelin is a peptidic hormone, which stimulates cell proliferation and inhibits apoptosis in several tissues, including pancreas. In preclinical stage of type 1 diabetes, proinflammatory cytokines generate a destructive environment for *β*-cells known as insulitis, which results in loss of *β*-cell mass and impaired insulin secretion, leading to diabetes. Our aim was to demonstrate that ghrelin could preserve *β*-cell viability, turnover rate, and insulin secretion acting as a counter balance of cytokines. In the present work we reproduced proinflammatory milieu found in insulitis stage by treating murine cell line INS-1E and rat islets with a cytokine cocktail including IL-1*β*, IFN*γ*, and TNF*α* and/or ghrelin. Several proteins involved in survival pathways (ERK 1/2 and Akt/PKB) and apoptosis (caspases and Bcl-2 protein family and endoplasmic reticulum stress markers) as well as insulin secretion were analyzed. Our results show that ghrelin alone has no remarkable effects on *β*-cells in basal conditions, but interestingly it activates cell survival pathways, downregulates apoptotic mediators and endoplasmic reticulum stress, and restores insulin secretion in response to glucose when beta-cells are cytokine-exposed. These data suggest a potential role of ghrelin in preventing or slowing down the transition from a preclinical to clinically established diabetes by ameliorating the effects of insulitis on *β*-cells.

## 1. Introduction

Ghrelin, a 28-amino acid peptidic hormone, is the endogenous ligand of the orphan receptor of growth hormone secretagogues (GHS-R). Its main functions are the stimulation of GH secretion by the pituitary somatotrophs [1] and appetite upregulation by inducing neuropeptide Y (NPY) production at the hypothalamus [2]. Besides, ghrelin regulates metabolism and energy balance at several levels [3].

Type 1 diabetes mellitus is an autoimmune disease in which pancreatic *β*-cells are specifically damaged—among other mechanisms—by apoptosis [4, 5]. Before the clinical onset of the disease, an inflammatory lesion known as insulitis develops due to the recruitment of macrophages and CD8+ T lymphocytes to the islets and the release of several soluble mediators and cytokines, mainly IFN-*γ*, TNF-*α*, and IL-1*β*, causing *β*-cells apoptosis [6]. This inflammatory microenvironment observed in pancreatic islets during type 1 diabetes can be mimicked by adding these cytokines to *β*-cell cultures.

Two main apoptotic pathways have been described: the intrinsic and the extrinsic pathways; both interconnected at several levels and both are activated by proinflammatory cytokines in pancreatic *β*-cells [7–9]. Endoplasmic reticulum (ER) stress-induced apoptosis has also been involved in cytokine toxicity to *β*-cells and cell lines, although it has not proven essential for the detrimental effects of cytokines [10–14]. In ER stress response, phosphorylation of the translation initiation factor EIF2A by EIF2AK3 is related with increased transcription of molecular chaperones such as HSPA4 and HSPA5 in an effort to prevent unfolded protein accumulation. The mitochondrial pathway of apoptosis is inhibited by the activation of some of the members of the Bcl family such as Bcl-2*α* and Bcl-xL, by preventing the release of proapoptotic mitochondrial factors [9]. Gene and protein expression of Bcl-2 related proteins have been studied in response to proinflammatory cytokines. Although results are identical for some of this protein family members such as Bid and Bcl-xL, contradictory results have been reported for others like Bcl-2; while Mehmeti et al. report a downregulation in its gene expression in response to cytokines, Kutlu et al. found that its levels were maintained in identical conditions. Bad gene expression also shows opposite results in these studies [15, 16].

Ghrelin's effects on *β*-cell proliferation and apoptosis have been extensively documented both “*in vitro*” and “*in vivo*” diabetes models. Ghrelin promotes proliferation and survival in insulin producing cell lines and “*ex vivo*” cultured human islets in presence of proinflammatory cytokines [17]. In the same line, ghrelin treatment recovers functional *β*-cell mass in pancreatectomized [18] or streptozotocin treated animals [19–21]. However, it has not been shown whether ghrelin has any effect on *β*-cells' insulin secretion in the presence of proinflammatory cytokines, which are well known to inhibit their insulin production in response to glucose [22].

The aim of this study is to elucidate how ghrelin acts on the apoptotic cascades to exert its protective effect on cytokine treated *β*-cell and if it restores *β*-cell function hindered by cytokine exposure. To this end, we reproduced an* in vitro *model of early insulitis in *β*-cells by using INS-1E [23] and Wistar rat islets cultured in the absence or presence of ghrelin and/or a cytokine cocktail, mimicking the cytotoxic conditions of pancreatic islets during early insulitis.

## 2. Materials and Methods

### 2.1. Cell Culture Materials

A well-established insulinoma cell line, INS-1E [23], was used in all* in vitro *experiments described below. INS-1E cells were routinely cultured in RPMI 1640 supplemented with 10% FBS, 1% Penicillin-Streptomycin, 1 mM sodium pyruvate (all from Gibco, Thermo Fisher Scientific, Cheshire, UK), 10 mM Hepes buffer in 0.85% NaCl (Lonza, Barcelona, Spain), 0.05 mM *β*-mercaptoethanol (Sigma-Aldrich), and 11 mM D-glucose (Merck, Darmstadt, Germany). For immunoblotting and flow cytometry experiments, 3 × 10^5^ cells were plated on 6-well dishes and were cultured until they reached 90% confluence.

To analyze the effects of cytokines, cell cultures were treated with a cytokine cocktail (100 ng/ml rat IFN-*γ*, 50 ng/ml rat TNF-*α*, and 0.05 ng/ml recombinant human IL1-*β*, all from Peprotech, Inc., London, UK) mimicking the cytotoxic conditions of preclinical diabetes. The cytokine treatment period was 15, 30, and 60 min for the ERK and AKT activation test and 48 h for cell viability study, Bcl-2 related protein expression quantification, ER stress, and the Glucose Stimulated Insulin Secretion (GSIS) determination. The effects of this cocktail have been previously described in cell cultures of pancreatic islets [24, 25].

To evaluate whether ghrelin reverses the deleterious effects of cytokines, 100 nM rat acylated ghrelin (Bachem AG, Bubendorf, Switzerland) was added to culture media alone and together with the cytokine cocktail. Ghrelin was renovated every 24 h. Ghrelin concentration was optimized previously in dose response experiments using INS-1E cells.

### 2.2. Trypan Blue Exclusion Test of Cell Viability

Dye exclusion tests are used to determine the rate of viable cells present in a cell suspension, based on the principle that live cells with intact cell membranes will exclude dyes, whereas dead cells will not. When our cell cultures in all different experimental conditions had reached a confluence of 90%, cell were collected and 10 microlitres of the suspension were mixed with the same volume of 0.2% trypan blue and then visually examined under an optical microscope on a Neubauer chamber to quantify viable cells.

### 2.3. MTT Assay

30000 cells/well were cultured in a 96-well plate at 37°C, 5% CO_2_. After 24 h, cultures were treated with different experimental conditions: control, cytokines, ghrelin 100 mM, and a mixture of cytokines and ghrelin during 48 h. Ghrelin was refreshed each 24 h. Culture supernatants were eliminated and 25 *μ*l of MTT solution diluted at 5 mg/mL (Merck Chemicals Limited, Darmstandt, Germany), was added to each well. Samples were incubated 90 minutes at 37°C with 5% CO_2_ and then washed with warm PBS. PBS was discarded and 100 *μ*l of DMSO/well was added. Plate was covered and gently shaked during 20 minutes at RT. Absorbance was measure at 570 nm.

### 2.4. Apoptosis Detection and Quantification

The rate of apoptosis was quantified by DeadEnd Fluorometric TUNEL System (TdT-mediated dUTP Nick-End Labeling) (Promega Corp., Madison, WI, USA) following the manufacturer's instructions. Briefly, samples were permeabilized with 0.2% Triton X-100/PBS for 20 min at RT, washed with PBS, and blocked with 4% FBS (Gibco) for 30 min. The samples were then equilibrated and incubated with the nucleotide mixture and the enzyme for 1 h at 37°C. The reaction was stopped with 2% SCC for 15 min at RT and then washed with water to eliminate fluorescein-12-dUTP excess. The results are expressed as the number of TUNEL-positive/total cells.

### 2.5. Isolation and Culture of Rat Islets

Pancreatic islets were isolated from adult male Wistar rats as described previously [26]. Islets were cultured in RPMI medium (Sigma-Aldrich, St. Louis, MO, USA) supplemented with 2 mM L-glutamine (Gibco Invitrogen, Carlsbad, CA, USA), 100 U/ml penicillin, 100 *μ*g/ml streptomycin (Pen-Strep; Bio-Whittaker Europe, Verviers, Belgium), 10% fetal bovine serum (FBS, Gibco Invitrogen, Carlsbad, CA, USA), and 5.5 mM glucose. Proinflammatory cytokines and ghrelin were used at the same concentrations described above.

### 2.6. Analysis of Glucose-Stimulated Insulin Secretion

To study the* in vitro* effect of ghrelin and/or cytokines on insulin secretion, INS-1E cells or isolated rat islets were cultured in 5.5 mM glucose medium for 24 h, to reduce basal insulin secretion. Subsequently, ghrelin with or without cytokines was added to culture media. Cells were then washed three times with low glucose (2.2 mM) HBSS/Krebs buffer (HBBS), and supernatants collected to determine basal insulin secretion. Afterwards, a high glucose concentration (22 mM) HBBS was added to cultures, and cells were incubated for 90 min. Supernatants were collected again to measure levels of glucose-induced insulin secretion.

Supernatants' insulin levels were determined using a commercially available Ultrasensitive Rat Insulin ELISA kit (Mercodia AB, Uppsala, Sweden) following manufacturer's instructions.

### 2.7. Immunoblotting

To determine the involvement of several proteins of the intrinsic and extrinsic apoptotic pathways in the response of INS-1E cells to ghrelin, cytokines, or both, western blot analysis was performed. First, cells were collected and centrifuged and pellets were homogenized in lysis buffer pH 6.8 (125 mM Tris, 2% SDS, 1 mM DTT, and Orthovanadate [1 : 100] containing the protease inhibitor cocktail) and supernatants protein concentration was measured using a Micro BCA kit (Thermo Fisher Scientific) following the manufacturer's instructions.

Protein samples (40 *μ*g) were loaded, electrophoresed on 10–15% SDS-PAGE, and transferred onto PVDF membranes (Immobilon-P, Millipore, EEUU). They were then blocked for 90 min at RT with 0.1% Tween-20, 5% (w/v) BSA/PBS. Afterwards, they were incubated overnight at 4°C with the primary antibodies for caspase-9 [1 : 1000] (Medical & Biological Laboratories Co., Naka-Ku Nagoya, Japan); caspase-8 [1 : 1000], Bax [1 : 1000] or Bcl-xL [1 : 1000] (Thermo Fisher Scientific), Bid [1 : 1000] (Abcam); Bcl-2 [1 : 1000] (Santa Cruz Biotechnology Inc., Heidelberg, Germany); phospho-Akt (Thr 308) [1 : 1000] and Akt [1 : 1000]; and phospho-ERK 1/2 [1 : 1000] and ERK 1/2 [1 : 1000] (Cell Signaling Technology Inc., Danvers, MA). Membranes were reprobed using a *β*-actin antibody [1 : 2000] (Abcam), as a loading control.

Membranes were subsequently washed for 3 times in 0.1% Tween-20/PBS and incubated with the corresponding secondary antibody conjugated with peroxidase [1 : 2000] in 0.1% Tween-20, 5% BSA-PBS, anti-rabbit IgG (Sigma-Aldrich, EEUU), and anti-mouse IgG (Sigma-Aldrich, EEUU), during 60 min at RT. Bound peroxidase activity was visualized by the enhanced chemiluminescence kit Immun-Star WesternC (BIO-RAD, EEUU) and quantified by densitometry using the analyzer Chemidoc XRS (BIO-RAD, EEUU) and Image J software (NIH, EEUU). Results were normalized to control values on each membrane.

### 2.8. Real-Time Reverse Transcription

Total RNA was isolated from INS-1E cells using the NucleoSpin RNA II Kit (Macherey-Nagel) followed by DNase treatment. cDNA was synthesized using the Transcriptor First Strand cDNA Synthesis kit (Roche). The real-time quantitative PCR reaction was performed from cDNA using SensiFAST SYBR No-ROX Kit (Bioline) according to the manufacturer's protocol (Bioline). The primer sets used are shown in Table 1. Amplification conditions were initial denaturation at 95°C for 3 min, final denaturation at 95°C for 15 s, annealing at 60°C for 20 s, and extension at 72°C for 20 s for 34 cycles, followed by melting curve analysis at temperature range of 60°C–90°C. Real-time data was acquired using Rotor-Gene 6000 (Corbett Research) real-time PCR detection system and analyzed using ΔΔCt method, with mRNA expression normalized.

### 2.9. Statistical Analysis

Results are presented as means + SEM of measurements performed in at least 4 independent experiments. Statistical comparisons were performed by Mann-Whitney test. Statistical significance was considered when *p* value was ≤0.05.

## 3. Results

### 3.1. Ghrelin Reduces Proinflammatory Cytokine Mediated Alterations of Survival Pathways in INS-1 Cells

**Figure 1:**
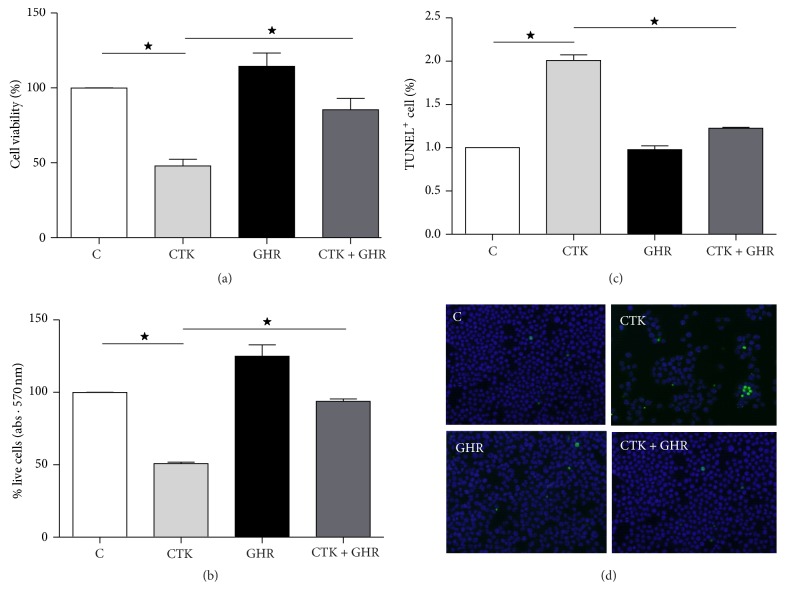
Ghrelin and cytokines' effects on cell survival and its survival pathways. Trypan blue exclusion test of cell viability (a), MTT assay for cell viability (b), and TUNEL-positive cells quantification to determine ghrelin's effects on apoptosis-mediated cellular death (c). Cells were incubated for 48 h in the absence or presence of cytokines, ghrelin, or both. (d) shows representative photomicrographs of control, cytokines (CTK), ghrelin (GHR), and cytokines + ghrelin (CTK + GHR), ^*∗*^ *p* < 0.05 between groups.

Firstly we tested if, in our model, we reproduced ghrelin's well-known effects on cell viability. To this end, we quantified viable cells in every culture condition at the time of collection of the samples by the trypan blue exclusion test and also performed an MTT assay. Cell viability was significantly reduced by the cytokine cocktail used in these experiments compared to control and ghrelin-treated cultures. When ghrelin was added together with cytokines, it significantly recovered INS-1E cells' viability (Figures 1(a) and 1(b)).

We then confirmed whether, as previously described, ghrelin would prevent cytokine-induced apoptosis in our cell line. In the first place, we performed a DeadEnd Fluorometric TUNEL assay which specifically detects DNA fragmentation resulting from apoptotic signaling cascades. The cytokine cocktail significantly increased INS-1E cell death after 48 h (Figure 1(c)) compared to controls and this effect was importantly reduced by incubation with ghrelin. The quantification of the number of TUNEL-positive cells versus total cells for each experimental condition showed that while cytokines increased apoptosis by 6-fold, coadministration of ghrelin reduced this percentage to nearly 50%.

**Figure 2:**
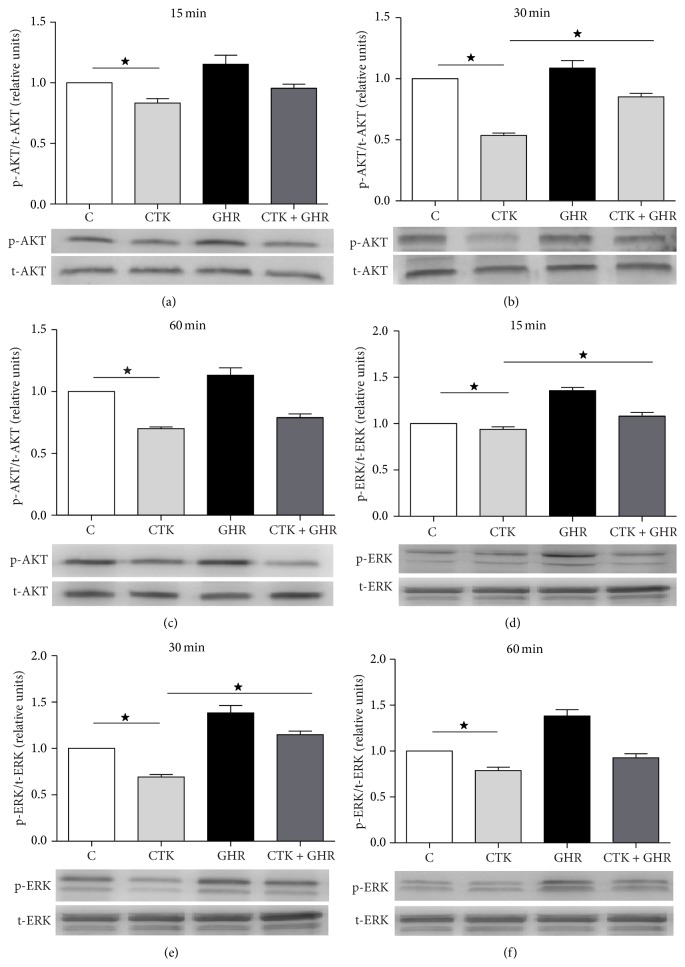
Western blot for phosphorylated AKT (a, c, and e) and ERK 1/2 (b, d, and f). Immunoblotting was performed to quantify phosphorylation level or AKT an ERK 1/2 treated with proinflammatory cytokines during 15 (a and d), 30 (b and e), and 60 min (c and f) alone or in presence of ghrelin. Panels below the graph show representative images of immunoblots. Results are expressed as mean ± SEM of phosphorylated to total protein ratio and referred to control cultures (c). Values were obtained from 4 independent experiments. ^*^ *p* < 0.05 between groups. C, control; CTK, cytokines; GHR, ghrelin; CTK + GHR, cytokines + ghrelin.

Akt/PKB is well known to be a key signaling protein in several cell pathways promoting survival, being frequently inactivated in response to a variety of stress stimuli [27]. Therefore, levels of Akt activation were investigated in our experimental model. Cytokine-treated cultures showed a significant reduction of phosphorylated Akt reaching its lowest at 30 min (Figures 2(a), 2(c), and 2(e)) compared to control or ghrelin-treated cultures. However, administration of ghrelin in addition to cytokines recovered Akt activation at 30 min, suggesting that ghrelin enhances the survival pathways promoted by Akt.

MAPK/ERK is another signaling pathway involved in proliferation and survival of pancreatic beta cells. To test if ghrelin modulates the effect of proinflammatory cytokines on ERK 1/2 activation, we examined phosphorylated ERK 1/2 levels by immunoblotting in each experimental condition. The results showed that cytokines reduced levels of active ERK 1/2 at 15, 30, and 60 min, while ghrelin significantly increased them at 15 and 30 min, compared to controls (Figures 2(b), 2(d), and 2(f)). Cell cultures receiving both cytokines and ghrelin showed activation levels similar to controls and significantly higher than those of cytokine-treated cultures, suggesting that ghrelin counteracts the toxicity of proinflammatory cytokines by inducing ERK 1/2 activation.

### 3.2. Ghrelin Restores Apoptotic Machinery Close to Basal Levels in Proinflammatory Cytokines Treated INS-1 Cells

**Figure 3:**
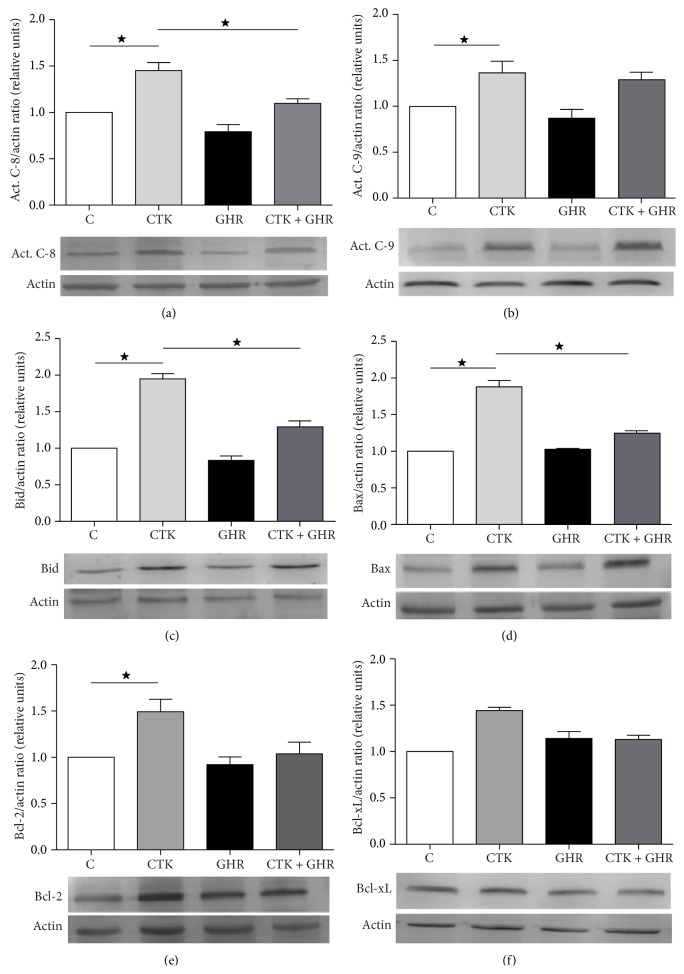
Ghrelin and cytokines' effects on levels of caspases and Bcl-2 protein family members. Immunoblotting was performed to measure relative mean levels of active caspase-8 (a); active caspase-9 (b); BID (c); Bax (d); Bcl-2 (e); and Bcl-xL (f). Panels below the graphs show representative images of studied protein immunoblots. Results are presented as mean ± SEM of actin/studied protein ratio and referred to control cultures (c). Values were obtained from 4 independent experiments. ^*^ *p* < 0.05 between groups. C, control; CTK, cytokines; GHR, ghrelin; CTK + GHR, cytokines + ghrelin.

To explore the cellular mechanisms involving ghrelin effects on proinflammatory cytokine mediated apoptosis of INS-1E cells, immunoblotting was performed to evaluate the upstream proteins involved in the intrinsic and extrinsic pathways of apoptosis, that is, caspase-9 and caspase-8, respectively. As previously reported, cytokine treatment induces caspase-8 and caspase-9 activation and ghrelin administration significantly prevented this effect in the case of caspase-8 (Figures 3(a) and 3(b)), suggesting that ghrelin inhibits the activation of the extrinsic pathway of apoptosis triggered by proinflammatory cytokines, probably upstream of caspase-8.

Furthermore, we analyzed the levels of several proteins of the Bcl-2 family. First, we studied the levels of the proapoptotic proteins BID and Bax. In our experimental model, cytokines significantly increased BID and Bax levels in INS-1E cells (Figures 3(c) and 3(d), resp.), and the addition of ghrelin to culture media prevented this effect.

Next, levels of the prosurvival members of the Bcl-2 family, Bcl-xL and Bcl-2*α*, were explored. Interestingly, the levels of Bcl-2*α* were also significantly increased in response to cytokine exposure compared to controls (Figure 3(e)). Ghrelin administration did not induce Bcl-2*α* upregulation, but when cells received ghrelin together with proinflammatory cytokines, the effect of the latter on Bcl-2*α* was completely attenuated, reaching control levels. Bcl-xL levels however did not significantly vary among different experimental conditions (Figure 3(f)).

### 3.3. Ghrelin Reduces Cytokines Mediated ER Stress Response Activation

**Figure 4:**
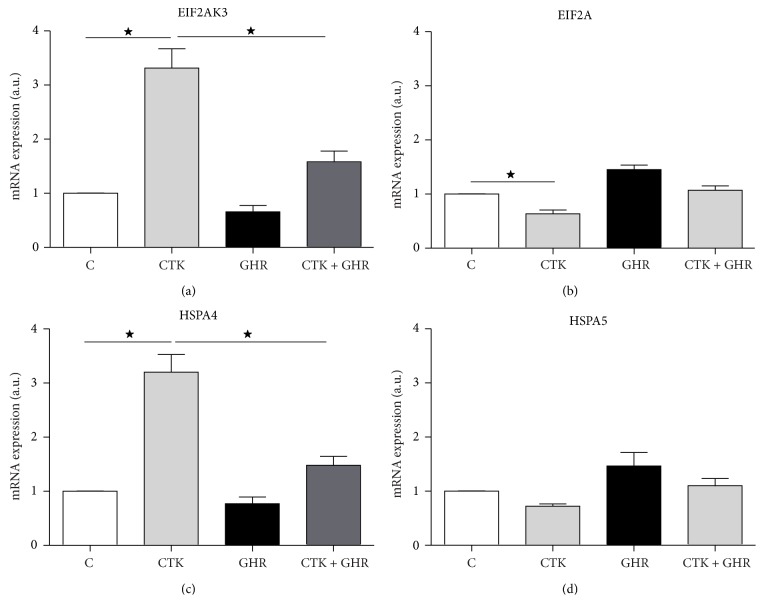
Ghrelin and cytokines' effects on ER stress response markers. Real-time quantitative PCR reaction was performed to measure gene levels of EIF2AK3 (a), EIF2A (b), HSPA4 (c), and HSPA5 (d) in cytokines treated cultures alone or in presence of ghrelin. Results are presented as mean ± SEM of mRNA expression indicated as arbitrary units. Values were obtained from 4 independent experiments. ^*^ *p* < 0.05 between groups. C, control; CTK, cytokines; GHR, ghrelin; CTK + GHR, cytokines + ghrelin.

To study effect of ghrelin in cytokines mediated ER stress response on INS-1 cells, gene expression of ER stress markers were determined. EIF2AK3 is an initiator of ER stress process and its expression is increased in response to cytokines. Ghrelin addition to cytokines treated cultures restores EIF2AK3 expression (Figure 4(a)). EIF2A, (EIF2AK3's substrate), slightly decreases its expression in response to cytokines and ghrelin treatment restores its level to control levels (Figure 4(b)). HSPA4 and HSPA5 chaperones expression was also quantified in response to cytokines alone or in combination with ghrelin and a 3-fold increase was observed with the addition of cytokines in HSPA4 mRNA expression (Figures 4(c) and 4(d)). This effect was reverted by ghrelin addition to cultures.

### 3.4. Ghrelin Recovers the Ability of *β*-Cells to Secrete Insulin in Response to Glucose Challenge under Cytotoxic Conditions

**Figure 5:**
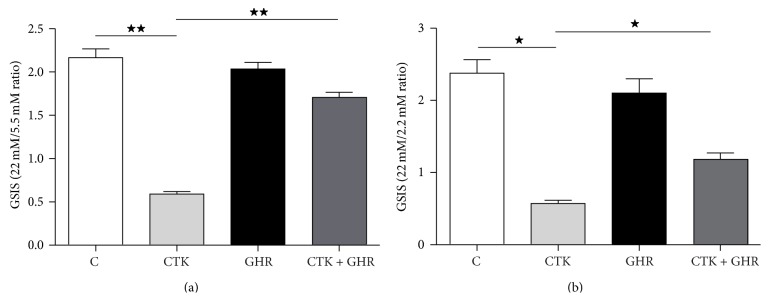
Ghrelin and cytokines' effects on insulin secretion. Glucose stimulated insulin secretion (GSIS) by INS-1 (a) and rat islet (b) cultures incubated in absence (C, control) or presence of cytokines (CTK), ghrelin (GHR), or both (CTK + GHR) for 48 h. Values are expressed as the ratio of insulin secreted at a stimulatory glucose concentration (22 mM) to insulin secreted at a basal glucose concentration (5.5 mM) and represent means ± SEM of values from 4 independent experiments. ^*^ *p* < 0.05; ^**^ *p* < 0.01 between groups.

INS-1E cells responded to the glucose overload challenge secreting insulin as expected, while the cytokine cocktail mimicking the proinflammatory condition of *β*-cells in a diabetic pancreas severely decreased their ability to produce insulin (Figure 5(a)). Importantly, ghrelin treatment of cytokines exposed cells recovered insulin secretion in response to glucose overload was by almost 75% compared to controls, suggesting that ghrelin could improve *β*-cell function during insulitis. Cultured rat islets exhibited a pattern of insulin secretion in response to glucose very similar to that of INS-1E cells in response to cytokines and ghrelin partially prevented this deleterious effect also (Figure 5(b)).

## 4. Discussion

The present study highlights the potential of ghrelin to protect pancreatic *β*-cells from cytokine-induced toxicity during insulitis, by activating survival pathways and by inhibiting the apoptosis machinery. A consequence of the molecular mechanisms promoted by ghrelin is the preservation of *β*-cell's insulin secretion in response to glucose when cultured with proinflammatory cytokines.

Cytokines are known to lead to important transcriptional and translational modifications both in INS-1 and INS-1E cells and in rat pancreatic islets that may eventually contribute to *β*-cell dysfunction and to cell death [16, 28–30]. Among these altered proteins, there are several involved in insulin secretion and in cell defense against endoplasmic reticulum and oxidative stress.

We reproduced in our model ghrelin's well-known beneficial effects on cell viability and then analyzed some signaling molecules involved in *β*-cell proliferation and survival. Levels of activated ERK 1/2 decreased at 15 and 30 min of cytokine exposure, explaining the decrease in cell proliferation previously reported by our group [24]. The same effect was observed when analyzing Akt/PKB activation levels; cytokines significantly decreased Akt/PKB activation levels at 30 min. This event which is in accordance with data from Li et al. [31] could be responsible for the reduced *β*-cell survival previously reported [17] in response to cytokines.

In our hands, ghrelin addition to cytokine-exposed *β*-cells completely reversed cytokine effects on ERK 1/2 activation at 15 and 30 min. A similar effect was reported in Doxorubicin-mediated apoptosis in HIT-T15 *β*-cell line [32]. In addition, ghrelin totally recovered phospho-Akt levels impaired by cytokines. These molecular events could be underlying the known effects of ghrelin on *β*-cell survival in cytotoxic models [17].

Cytokines are known to trigger *β*-cell apoptosis by two main mechanisms: activating proapoptotic signaling pathways, such as NF*κ*B [33], and promoting the activity of inducible nitric oxide synthase (iNOS) [34]. Thus, we confirmed that cytokines induced apoptosis in our model and this effect occurred by caspase-8 and caspase-9 activation, triggering both the extrinsic and intrinsic pathways of apoptosis as previously described by others [8, 35, 36]. Ghrelin addition recovered this effect by inhibiting caspase-8 activation. Absence of caspase-9 activation reversion suggests a downstream effect of ghrelin on intrinsic pathway.

In addition, when analyzing the mitochondrial pathway of apoptosis, in which the Bcl-2 protein family is involved, we observed the activation of the proapoptotic “BH3-only” member Bid, as well as the multidomain member Bax in response to cytokines. Bid and Bax may act as the intermediaries between extrinsic and intrinsic apoptosis pathways. Indeed, we found an upregulation of the whole pathway, justifying the increased levels of active caspase-3 reported by others [17]. These data are in accordance with an analysis of the gene networks affected by cytokine treatment in INS-1E cells [16]. We found that ghrelin was capable of restoring this pathway to control levels, protecting INS-1E cells against apoptotic cell death, as other authors have reported in different cell lines [17, 32, 37].

We also studied the levels of the antiapoptotic members of Bcl-2 protein family, that is, Bcl-2*α* and Bcl-X_L_. We observed no differences in Bcl-X_L_ protein levels in any of the experimental conditions. Surprisingly, Bcl-2*α* protein levels show a significant increment in response to cytokine treatment. This observed Bcl-2 increment could be a compensatory mechanism to counteract apoptotic stimulus. These findings are not in accordance with those described by Mehmeti et al. although this difference could be explained by the experimental designs (different cell lines and time of cytokines exposure) [15]. In contrast, the coadministration of ghrelin to these cultures normalized Bcl-2*α* levels to those found in control conditions, confirming that this hormone interferes with proapoptotic mechanisms and promotes cell survival in our experimental system [17, 38].

Another pathway implicated in proinflammatory cytokine induced beta cell apoptosis is the ER stress response. In INS-1 cells, ghrelin role in ER stress response modulation has been studied by determining gene expression levels of ER stress markers such us EIF2AK3, EIF2A, HSPA4, and HSPA5. Cytokines induce an increment of EIF2AK3 and HSPA4 expression (Figures 3(a) and 3(c)), while ghrelin prevents this effect, suggesting that ghrelin participates also in the regulation of ER stress response. The absence of cytokine effect in EIF2A gene expression could be because its regulation is mainly by phosphorylation [39].

It is important to highlight that ghrelin has shown no effect on basal activation of ERK, Akt, or caspases at the doses used. This issue is crucial when considering the safety, when considering a potential use of ghrelin in *β*-cell preservation strategies.

Finally, we would like to pinpoint that ghrelin did not affect insulin secretion in this cell line in basal conditions, in accordance with* in vivo* models [40], and most importantly, it recovered INS-1E cells' ability to secrete insulin in response to glucose under cytotoxic conditions. These results were confirmed in rat islets primary cultures, suggesting that ghrelin treatment could improve glucose homeostasis during the insulitis phase of diabetes development. Some authors have described an* in vivo* negative effect of ghrelin on insulin secretion; however these studies used higher doses of the hormone [41–44]. Nevertheless, several authors have demonstrated that ghrelin may restore glucose homeostasis following streptozotocin *β*-cell injury [45]. Our data encourage studying the* in vivo* mechanisms of ghrelin in the pancreas regarding apoptosis, cell proliferation, and insulin secretion in animal models of autoimmune diabetes mellitus.

## 5. Conclusions

Our results show that ghrelin alone does not affect any of the parameters we have analyzed in relation to survival and apoptosis or glucose homeostasis as compared to controls, suggesting that it does not affect basal *β*-cell turnover and strengthening the idea that its therapeutic use would be safe regarding these functions.

Moreover, ghrelin restores proinflammatory cytokines induced alteration of several proteins involved in *β*-cell apoptosis and survival pathways and increases the ability of both INS-1E cells and islets in primary culture to secrete insulin in response to glucose under cytotoxic conditions. Taken together, our data support a potential therapeutic use of ghrelin as a *β*-cell mass and function preserving factor under cytotoxic conditions.

## Figures and Tables

**Table 1:** Primer sets used in real-time quantitative PCR determinations.

Gene symbol	Alias/common name	Primer sequence (5′-nyt-3′)	Product size (bp)	Annealing temperature (°C)
HSPA4	Hsp70, Heat Shock 70 KDa protein 4 (a molecular chaperone involved in ER stress response)	F-AGCAAGCGCTCTCGGTTGCAG	133	60
		R-AGACAGGACACGGACCCCCG		

HSPA5	Bip, Heat Shock 70 KDa protein 5 (a molecular chaperone involved in ER stress response)	F-TGCTGCTGCCCAACTGGCTG	160	60
		R-GAACACGCCGACGCAGGAGT		

EIF2A	Eukaryotic translation initiation factor 2A	F-ACGCCGCTCTTGACAGTCCG	152	60
		R-TTGCCCCAGGCAAACAAGGTCC		

EIF2AK3	Eukaryotic translation initiation factor 2 alpha kinase 3	F-CCCCAACAAGGCCAGCCTGG	168	60
		R-GGACAGCCAGCCGTGTTCCC		

## References

[1] Kojima M., Hosoda H., Date Y., Nakazato M., Matsuo H., Kangawa K. (1999). Ghrelin is a growth-hormone-releasing acylated peptide from stomach. *Nature*.

[2] Inui A., Asakawa A., Bowers C. Y. (2004). Ghrelin, appetite, and gastric motility: the emerging role of the stomach as an endocrine organ. *The FASEB Journal*.

[3] Sato T., Nakamura Y., Shiimura Y., Ohgusu H., Kangawa K., Kojima M. (2012). Structure, regulation and function of ghrelin. *Journal of Biochemistry*.

[4] Bottazzo G., Florin-Christensen A., Doniach D. (1974). Islet-cell antibodies in diabetes mellitus with autoimmune polyendocrine deficiencies. *The Lancet*.

[5] Maccuish A. C., Irvine W. J., Barnes E. W., Duncan L. J. P. (1974). Antibodies to pancreatic islet cells in insulin-dependent diabetics with coexistent autoimmune disease. *The Lancet*.

[6] Eizirik D. L., Colli M. L., Ortis F. (2009). The role of inflammation in insulitis and beta-cell loss in type 1 diabetes. *Nature Reviews Endocrinology*.

[7] Cottet S., Dupraz P., Hamburger F., Dolci W., Jaquet M., Thorens B. (2002). cFLIP protein prevents tumor necrosis factor-*α*-mediated induction of caspase-8-dependent apoptosis in insulin-secreting *β*Tc-Tet cells. *Diabetes*.

[8] Grunnet L. G., Aikin R., Tonnesen M. F. (2009). Proinflammatory cytokines activate the intrinsic apoptotic pathway in *β*-cells. *Diabetes*.

[9] Kantari C., Walczak H. (2011). Caspase-8 and bid: caught in the act between death receptors and mitochondria. *Biochimica et Biophysica Acta*.

[10] Åkerfeldt M. C., Howes J., Chan J. Y. (2008). Cytokine-induced *β*-cell death is independent of endoplasmic reticulum stress signaling. *Diabetes*.

[11] Vasu S., McClenaghan N. H., McCluskey J. T., Flatt P. R. (2014). Mechanisms of toxicity by proinflammatory cytokines in a novel human pancreatic beta cell line, 1.1B4. *Biochimica et Biophysica Acta—General Subjects*.

[12] Cnop M., Welsh N., Jonas J.-C., Jörns A., Lenzen S., Eizirik D. L. (2005). Mechanisms of pancreatic *β*-cell death in type 1 and type 2 diabetes: many differences, few similarities. *Diabetes*.

[13] Kharroubi I., Ladrière L., Cardozo A. K., Dogusan Z., Cnop M., Eizirik D. L. (2004). Free fatty acids and cytokines induce pancreatic *β*-cell apoptosis by different mechanisms: role of nuclear factor-*κ*B and endoplasmic reticulum stress. *Endocrinology*.

[14] Cardozo A. K., Ortis F., Storling J. (2005). Cytokines downregulate the sarcoendoplasmic reticulum pump Ca^2+^ ATPase 2b and deplete endoplasmic reticulum Ca^2+^, leading to induction of endoplasmic reticulum stress in pancreatic *β*-cells. *Diabetes*.

[15] Mehmeti I., Lenzen S., Lortz S. (2011). Modulation of Bcl-2-related protein expression in pancreatic beta cells by pro-inflammatory cytokines and its dependence on the antioxidative defense status. *Molecular and Cellular Endocrinology*.

[16] Kutlu B., Cardozo A. K., Darville M. I. (2003). Discovery of gene networks regulating cytokine-induced dysfunction and apoptosis in insulin-producing INS-1 cells. *Diabetes*.

[17] Granata R., Settanni F., Biancone L. (2007). Acylated and unacylated ghrelin promote proliferation and inhibit apoptosis of pancreatic *β*-cells and human islets: involvement of 3′,5′-cyclic adenosine monophosphate/protein kinase A, extracellular signal-regulated kinase 1/2, and phosphatidyl inositol 3-kinase/Akt signaling. *Endocrinology*.

[18] Kerem M., Salman B., Ozsoy S. (2009). Exogenous ghrelin enhances endocrine and exocrine regeneration in pancreatectomized rats. *Journal of Gastrointestinal Surgery*.

[19] Irako T., Akamizu T., Hosoda H. (2006). Ghrelin prevents development of diabetes at adult age in streptozotocin-treated newborn rats. *Diabetologia*.

[20] Granata R., Volante M., Settanni F. (2010). Unacylated ghrelin and obestatin increase islet cell mass and prevent diabetes in streptozotocin-treated newborn rats. *Journal of Molecular Endocrinology*.

[21] Granata R., Settanni F., Julien M. (2012). Des-acyl ghrelin fragments and analogues promote survival of pancreatic *β*-cells and human pancreatic islets and prevent diabetes in streptozotocin-treated rats. *Journal of Medicinal Chemistry*.

[22] Corbett J. A., McDaniel M. L. (1995). Intraislet release of interleukin 1 inhibits *β* cell function by inducing *β* cell expression of inducible nitric oxide synthase. *Journal of Experimental Medicine*.

[23] Merglen A., Theander S., Rubi B., Chaffard G., Wollheim C. B., Maechler P. (2004). Glucose sensitivity and metabolism-secretion coupling studied during two-year continuous culture in INS-1E insulinoma cells. *Endocrinology*.

[24] Blandino-Rosano M., Perez-Arana G., Mellado-Gil J. M., Segundo C., Aguilar-Diosdado M. (2008). Anti-proliferative effect of pro-inflammatory cytokines in cultured *β* cells is associated with extracellular signal-regulated kinase 1/2 pathway inhibition: protective role of glucagon-like peptide-1. *Journal of Molecular Endocrinology*.

[25] Mellado-Gil J. M., Aguilar-Diosdado M. (2004). High glucose potentiates cytokine- and streptozotocin-induced apoptosis of rat islet cells: effect on apoptosis-related genes. *Journal of Endocrinology*.

[26] McDaniel M. L., Colca J. R., Kotagal N., Lacy P. E. (1983). A subcellular fractionation approach for studying insulin release mechanisms and calcium metabolism in islets of Langerhans. *Methods in Enzymology*.

[27] Lindsley C. W. (2010). The Akt/PKB family of protein kinases: a review of small molecule inhibitors and progress towards target validation: a 2009 update. *Current Topics in Medicinal Chemistry*.

[28] Cardozo A. K., Kruhøffer M., Leeman R., Ørntoft T., Eizirik D. L. (2001). Identification of novel cytokine-induced genes in pancreatic *β*-cells by high-density oligonucleotide arrays. *Diabetes*.

[29] D'Hertog W., Overbergh L., Lage K. (2007). Proteomics analysis of cytokine-induced dysfunction and death in insulin-producing INS-1E cells: new insights into the pathways involved. *Molecular and Cellular Proteomics*.

[30] Lopes M., Kutlu B., Miani M. (2014). Temporal profiling of cytokine-induced genes in pancreatic *β*-cells by meta-analysis and network inference. *Genomics*.

[31] Li L., El-Kholy W., Rhodes C. J., Brubaker P. L. (2005). Glucagon-like peptide-1 protects beta cells from cytokine-induced apoptosis and necrosis: role of protein kinase B. *Diabetologia*.

[32] Zhang Y., Ying B., Shi L. (2007). Ghrelin inhibit cell apoptosis in pancreatic *β* cell line HIT-T15 via mitogen-activated protein kinase/phosphoinositide 3-kinase pathways. *Toxicology*.

[33] Ortis F., Cardozo A. K., Crispim D., Störling J., Mandrup-Poulsen T., Eizirik D. L. (2006). Cytokine-induced proapoptotic gene expression in insulin-producing cells is related to rapid, sustained, and nonoscillatory nuclear factor-*κ*B activation. *Molecular Endocrinology*.

[34] Størling J., Binzer J., Andersson A. K. (2005). Nitric oxide contributes to cytokine-induced apoptosis in pancreatic beta cells via potentiation of JNK activity and inhibition of Akt. *Diabetologia*.

[35] Santin I., Moore F., Colli M. L. (2011). PTPN2, a candidate gene for type 1 diabetes, modulates pancreatic *β*-cell apoptosis via regulation of the BH3-only protein bim. *Diabetes*.

[36] Moore F., Santin I., Nogueira T. C. (2012). The transcription factor C/EBP delta has anti-apoptotic and anti-inflammatory roles in pancreatic beta cells. *PLoS ONE*.

[37] Wang W., Zhang D., Zhao H. (2010). Ghrelin inhibits cell apoptosis induced by lipotoxicity in pancreatic *β*-cell line. *Regulatory Peptides*.

[38] Zhou X., Xue C. (2009). Ghrelin inhibits the development of acute pancreatitis and nuclear factor kappaB activation in pancreas and liver. *Pancreas*.

[39] Pavitt G. D., Ron D. (2012). New insights into translational regulation in the endoplasmic reticulum unfolded protein response. *Cold Spring Harbor Perspectives in Biology*.

[40] Bando M., Iwakura H., Ariyasu H. (2012). Transgenic overexpression of intraislet ghrelin does not affect insulin secretion or glucose metabolism in vivo. *The American Journal of Physiology—Endocrinology and Metabolism*.

[41] Wren A. M., Small C. J., Abbott C. R. (2001). Ghrelin causes hyperphagia and obesity in rats. *Diabetes*.

[42] Dezaki K., Sone H., Yada T. (2008). Ghrelin is a physiological regulator of insulin release in pancreatic islets and glucose homeostasis. *Pharmacology and Therapeutics*.

[43] Sangiao-Alvarellos S., Cordido F. (2010). Effect of ghrelin on glucose-insulin homeostasis: therapeutic implications. *International Journal of Peptides*.

[44] Cordido F. (2010). Insulin regimens in type 2 diabetes. *The New England Journal of Medicine*.

[45] Bando M., Iwakura H., Ariyasu H. (2013). Overexpression of intraislet ghrelin enhances *β*-cell proliferation after streptozotocin-induced *β*-cell injury in mice. *The American Journal of Physiology—Endocrinology and Metabolism*.

